# Green synthesis and antibacterial applications of gold and silver nanoparticles from *Ligustrum vulgare* berries

**DOI:** 10.1038/s41598-022-11811-7

**Published:** 2022-05-12

**Authors:** Priyanka Singh, Ivan Mijakovic

**Affiliations:** 1 The Novo Nordisk Foundation, Center for Biosustainability, Technical University of Denmark, 2800 Kogens Lyngby, Denmark; 2 Systems and Synthetic Biology Division, Department of Biology and Biological Engineering, Chalmers University of Technology, 412 96 Gothenburg, Sweden

**Keywords:** Microbiology, Materials science, Nanoscience and technology

## Abstract

Increasing demand for green or biological nanoparticles has led to various green technologies and resources, which play a critical role in forming biocompatible or green nanoparticles. So far, numerous medicinal plants have been explored for this purpose, assuming that medicinal components from the plant's material will contribute to corona formation around nanoparticles and enhance their efficacy. Research is also extended to other green and waste resources to be utilized for this purpose. In the current study, we explored *Ligustrum vulgare* berries, also known as privet berries, to reduce gold and silver salts into nanoparticles. *L. vulgare* berries showed great potential to form these nanoparticles, as gold nanoparticles (LV-AuNPs) formed within 5 min at room temperature, and silver nanoparticles (LV-AgNPs) formed in 15 min at 90 °C. LV-AuNPs and LV-AgNPs were characterized by various analytical methods, including UV–Vis, SEM, EDX, TEM, DLS, sp-ICP-MS, TGA, FT-IR, and MALDI-TOF. The results demonstrate that the LV-AuNPs are polydisperse in appearance with a size range 50–200 nm. LV-AuNPs exhibit various shapes, including spherical, triangular, hexagonal, rod, cuboid, etc. In contrast, LV-AgNPs are quite monodisperse, 20–70 nm, and most of the population was spherical. The nanoparticles remain stable over long periods and exhibit high negative zeta potential values. The antimicrobial investigation of LV-AgNPs demonstrated that the nanoparticles exhibit antibacterial ability with an MBC value of 150 g/mL against *P. aeruginosa* and 100 g/mL against *E. coli*, as determined by plate assay, live and dead staining, and SEM cell morphology analysis.

## Introduction

The global search for suitable methods for the generation of green nanoparticles leads to the rapid development of new technologies and discovery of new resources for nanoparticles synthesis. Especially, there is a high demand for green methods that can produce nanoparticles in an eco-friendly manner and maintain long-term stability. Green synthesis is also catching attention because of the corona layer formation surrounding the nanoparticles. In green synthesis, the source (biomaterial) constituents help reduce metal salts to nanoparticles and play a major role in stabilizing nanoparticles by forming a capping layer that surrounds the nanoparticles called the “biological corona”. In addition to providing stability, the biological corona contributes to nanoparticles' biocompatibility and affects their mode of action^1^.

The simplest way to achieve green nanoparticles is to use biological resources, such as microorganisms, plants, or their parts, for nanoparticle production. So far, numerous microorganisms have been explored for this purpose and found efficient in green nanoparticles synthesis, with synthesis occurring either extra or intracellularly, with monodispersity and long-term stability of nanoparticles^2,3^. However, there are certain limitations with microbial production, such as the demand for specific growth conditions, culture incubators that use energy resources, complex purification systems that require several cycles of high-speed centrifugation, washing, and sometimes ultrasonication to break the membrane for collecting intracellular nanoparticles^4^. These limitations can be overcome in green synthesis by utilizing plants. Various plants have been found suitable for the rapid production of facile, eco-friendly nanoparticles without energy consumption. Interestingly, plant-based nanoparticle production typically happens within a few seconds to minutes. In addition, plant-derived nanoparticles are quite stable, biocompatible, and monodisperse^5^. Especially, medicinal plants are very popular in this sense due to the attachment of medicinal components from plant extract to the nanoparticles corona, which could enhance the nanoparticles' efficiency for certain applications^6–8^. In addition, the waste products of plants, such as e.g. onion peels, can be used for nanoparticles synthesis, which is an especially good and sustainable production setup^9,10^. To expand on available venues for the synthesis of green nanoparticles, we used *L. vulgare* berries to prepare an aqueous extract, which was used to form stable gold and silver nanoparticles. *L. vulgare* is also called a common pivet or European pivet; it is a semi-evergreen and deciduous shrub. It usually grows up to 3–4 m and can be found in Europe, North Africa, and Asia. In Scandinavian countries, *L. vulgare* plants are grown in large amounts to make green hedges. The berries of these plants are blueish black and available in large amounts during spring. These berries germinate every year, but they are not used for food or other purposes, so they simply dry out on plants and fall off. Our study showed the possibility of using these otherwise waste and widely available biological resources to produce highly-cost products: gold and silver nanoparticles.

We chose gold and silver nanoparticles due to their high demand and numerous application in the biomedical field^11,12^. Gold nanoparticles are highly utilized for medical purposes like diagnostic, sensors development, photo imaging, and photothermal therapeutic applications^13–15^. Silver nanoparticles also showed immense potential for many applications in different fields such as in textile, packaging, shipping, medical, and cosmetics due to their antimicrobial property^16^. The antimicrobial property of silver nanoparticles has made them the first choice for various applications in which microbial contamination is a major concern. For instance, in medical devices, treating wound infections, packaging, textiles, medical device and disinfectants, etc.^17,18^. The high performance of silver nanoparticles creates a high demand for their production. Thus, The current study explored the *L. vulgare* berries as a green resource for gold and silver nanoparticles formation. We used the widely available but wasted resources to produce a high-value product.. In addition to a thorough analytical characterization for both nanoparticle types, we explored the *L. vulgare* berries mediated silver nanoparticles (LV-AgNPs) for antimicrobial application against two Gram-negative pathogens: *P. aeruginosa* and *E. coli*.

## Materials and methods

### Materials

Analytical grade gold (III) chloride trihydrate (HAuCl_4_·3H_2_O) and silver nitrate (AgNO_3_) were purchased from Sigma–Aldrich Chemicals (St Louis, MO, USA). To prepare the aqueous extract of L. vulgare berries, we used 10 g of fresh berries and boiled them with 90 mL of distilled water. The boiling mixture was first filtered and then centrifuged at 8000 rpm for 5 min to remove any suspended particulates^19,20^. The aqueous extract was diluted with different water ratios and referred to as a synthesis medium (SM).

### Green synthesis of LV-AuNPs and LV-AgNPs

For the nanoparticle's synthesis, a previously reported methodology was followed^20^. We used the whole extract as a synthesis medium (SM) without dilution with 1 mM of gold salt (HAuCl_4_·3H_2_O) and silver salt (AgNO_3_) to produce LV-AuNPs and LV-AgNPs, respectively. Once the synthesis was confirmed for LV-AuNPs at room temperature and LV-AgNPs at 90 ℃, we determined the optimization factors for nanoparticles formation. For optimization studies, we used multiple dilutions of extract to autoclave water at different temperatures and ranges of salt concentrations. The optimized parameters were determined based on visual inspection and UV–Vis spectrum of formed nanoparticles.

### Analytical characterization of LV-AuNPs and LV-AgNPs

UV–Vis, SEM, TEM, DLS, ICPMS, TGA, and MALDI-TOF studies were done as reported before^5^. Pure nanoparticles were suspended in water used for analytical characterization and application. For thermogravimetric analysis (TGA) and Fourier Transform-Infrared Spectroscopy (FT-IR), nanoparticles were air-dried to form a pellet.

### Antibacterial activity of LV-AgNPs

The antimicrobial activity of LV-AgNPs was evaluated against two Gram-negative pathogens: *Escherichia coli* UTI 89, and *Pseudomonas aeruginosa* PAO1. The antimicrobial study was conducted as reported before^21^. Live and dead staining and SEM microscopic observation of dead and control cells were also performed as described before^2^.

## Results

### Formation of LV-AuNPs and LV-AgNPs

**Figure 1:**
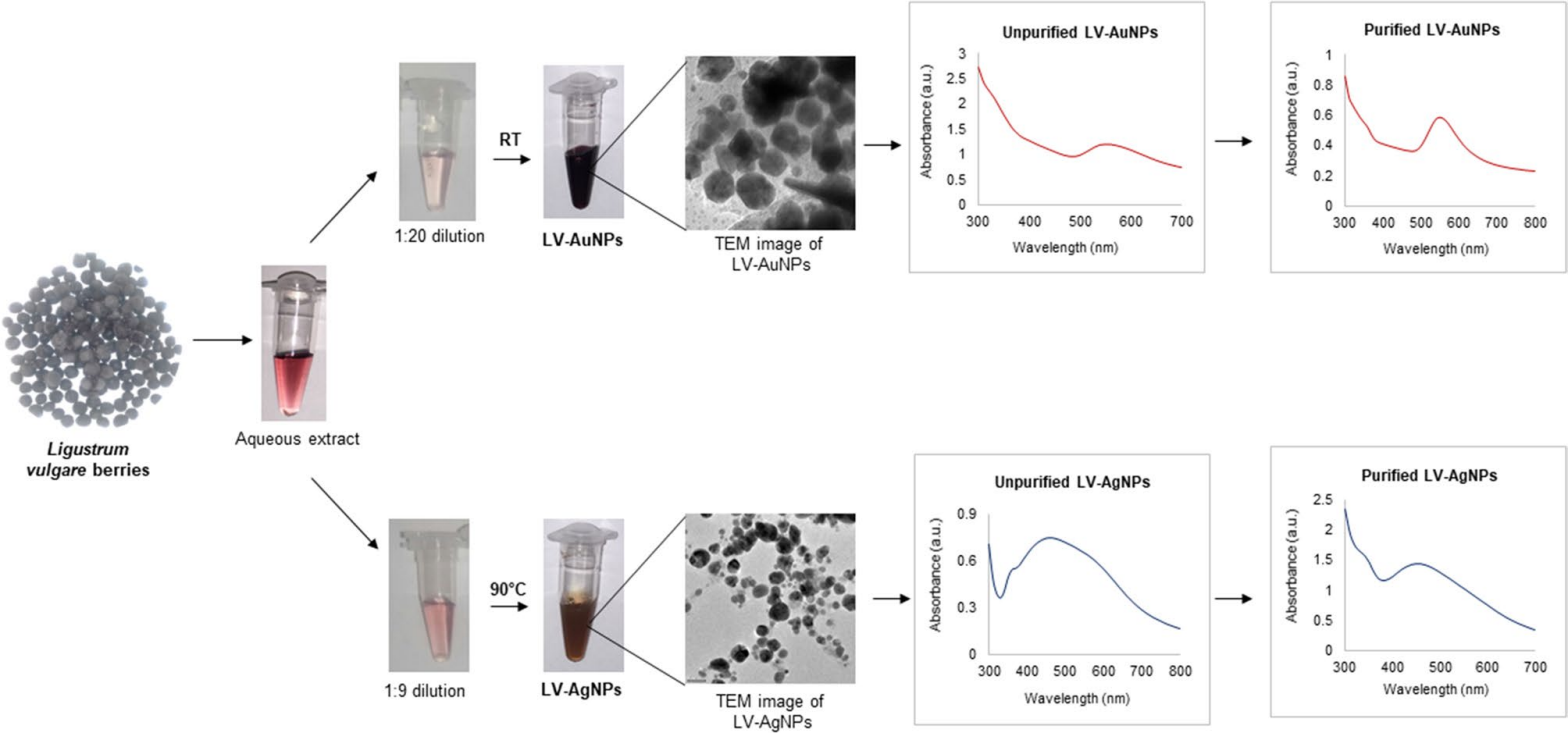
Schematic representation and UV-vis spectra of LV-AuNPs and LV-AgNPs formation form aqueous extract of L. vulgare berries.

**Figure 2:**
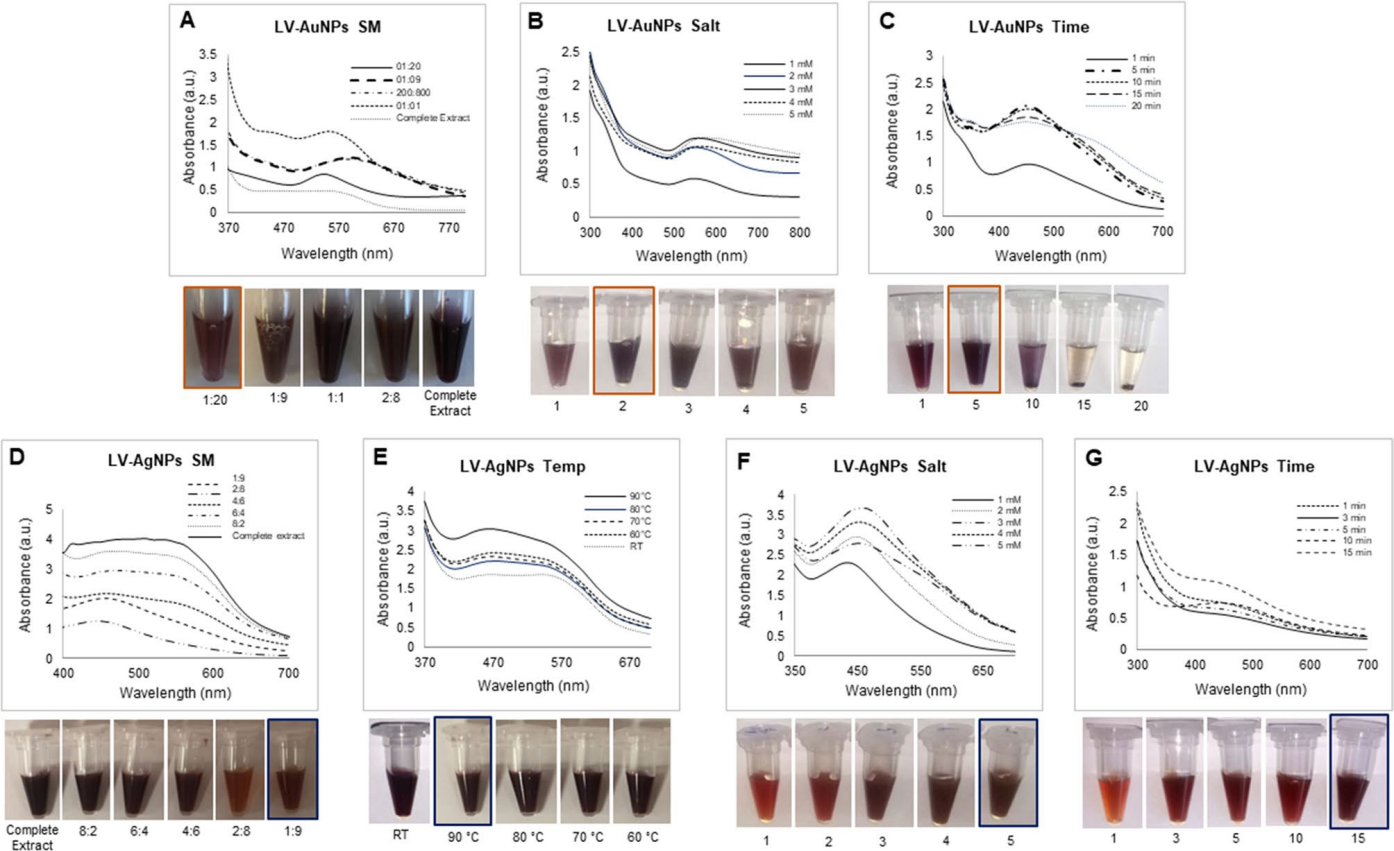
Optimization studies for LV-AuNPs and LV-AgNPs production. For LV-AuNPs production optimization with visible picture and UV-Vis spectrum, (A) synthesis medium; (B) gold salt optimization; (C) time optimization. For LV-AgNPs production optimization with visible picture and UV-Vis spectrum, (D) synthesis medium, (E) temperature, (F) silver slat concentration and (G) synthesis time.

Visual inspection and UV–Vis spectra were used to track the formation of LV-AuNPs and LV-AgNPs. The aqueous extract of *L. vulgare* berries was discovered to reduce gold and silver salt to LV-AuNPs and LV-AgNPs, respectively (Fig. 1). A change in color of the SM, which is a mixture of *L. vulgare* berries aqueous extract, and salt solution, verified the synthesis. From the light pink color of the *L. vulgare* berries extract, the color of LV-AuNPs changed to dark purple, while the color of LV-AgNPs changed to brown. This color change correlates to the surface plasmon resonance (SPR) feature of the produced nanoparticles. A UV–Vis spectrum of samples in the region of 300–700 nm was used to confirm the synthesis. A defined peak was observed in the range of 500–600 nm^22^, for LV-AuNPs, and a maximum and broad peak was observed in the 400–500 nm for LV-AgNPs region^23^. After three rounds of centrifugation and DI water washing, the nanoparticle samples were scanned with UV–Vis spectroscopy. The data revealed a high-intensity peak in a similar region (Fig. 1). The optimization studies for LV-AuNPs and LV-AgNPs were also conducted by using UV–Vis spectrum analysis and visible observations. For LV-AuNPs, the optimal ratio of SM was 1:20 (Fig. 2A), HAuCl_4_·3H_2_O concentration was 2 mM (Fig. 2B), and the reaction took place at room temperature within 5 min. For LV-AgNPs, the optimal ratio of SM for nanoparticles production was 1:9 (Fig. 2D), temperature 90 °C (Fig. 2E), 5 mM silver salt concentration (Fig. 2F), and synthesis time noted was 15 min (Fig. 2G).

### Characterization of LV-AuNPs and LV-AgNPs

**Figure 3:**
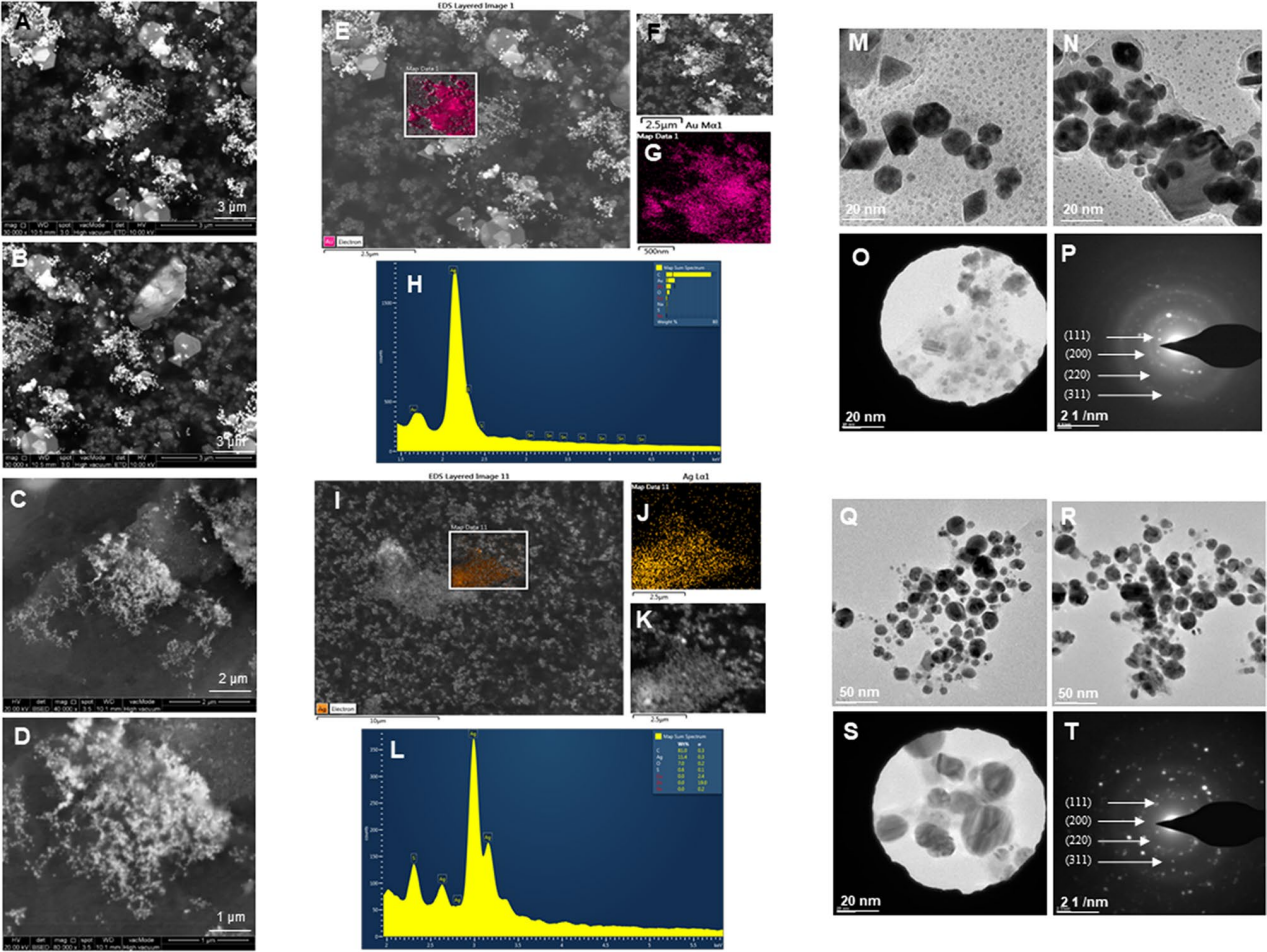
Structural analysis of LV-AuNPs and LV-AgNPs. For LV-AuNPs, (A, B) SEM images of nanoparticles at different scales, (E–G) Elemental mapping of LV-AuNPs showing scanned image of NPs with gold element distribution (pink), (H) EDX spectrum of the elemental mapped region showing sharp peak for gold element. (M,N) TEM image of LV-AuNPs, (O,P) SAED pattern. For LV-AgNPs, (C,D) SEM images of nanoparticles at different scales, (I-K) Elemental mapping of LV-AgNPs showing scanned image of NPs with silver element distribution (orange), (L) EDX spectrum of the elemental mapped region showing highest peak for silver element. (Q,R) TEM image of LV-AgNPs, (S,T) SAED pattern of LV-AgNPs.

To investigate the detailed morphological features of LV-AuNPs and LV-AgNPs, the purified and concentrated samples of nanoparticles were studied by SEM, EDX, elemental mapping, TEM, and SAED. SEM displayed the spherical, triangular, hexagonal rods; cuboid shapes for LV-AuNPs, which cause polydispersities (Fig. 3A,B). In contrast, for LV-AgNPs, we found a spherical form in the majority of the population (Fig. 3C,D). In addition, we conducted EDX, and elemental mapping, which showed a clear map of gold and silver elements in LV-AuNPs (Fig. 3E–G) and LV-AgNPs scanned micrographs (Fig. 3I–K). EDX displayed the clear peak of gold and silver elements, confirming the maximum distribution of gold (Fig. 3H) and silver elements (Fig. 3L) in the respective samples^24^. TEM image analysis revealed the different shapes and confirmed that an organic layer derived from the berries extract covers the nanoparticles. In addition, TEM analysis also revealed the different shapes for LV-AuNPs with a core diameter of 50–200 nm (Fig. 3M,N) and LV-AgNPs with size 20–70 nm (Fig. 3Q,R), respectively. The SAED pattern corresponds to the main reflection lattice planes of (111), (200), (220), and (311), which represents the polycrystalline nature of LV-AuNPs (Fig. 3O,P) and LV-AgNPs (Fig. 3S,T)^25,26^.

**Figure 4:**
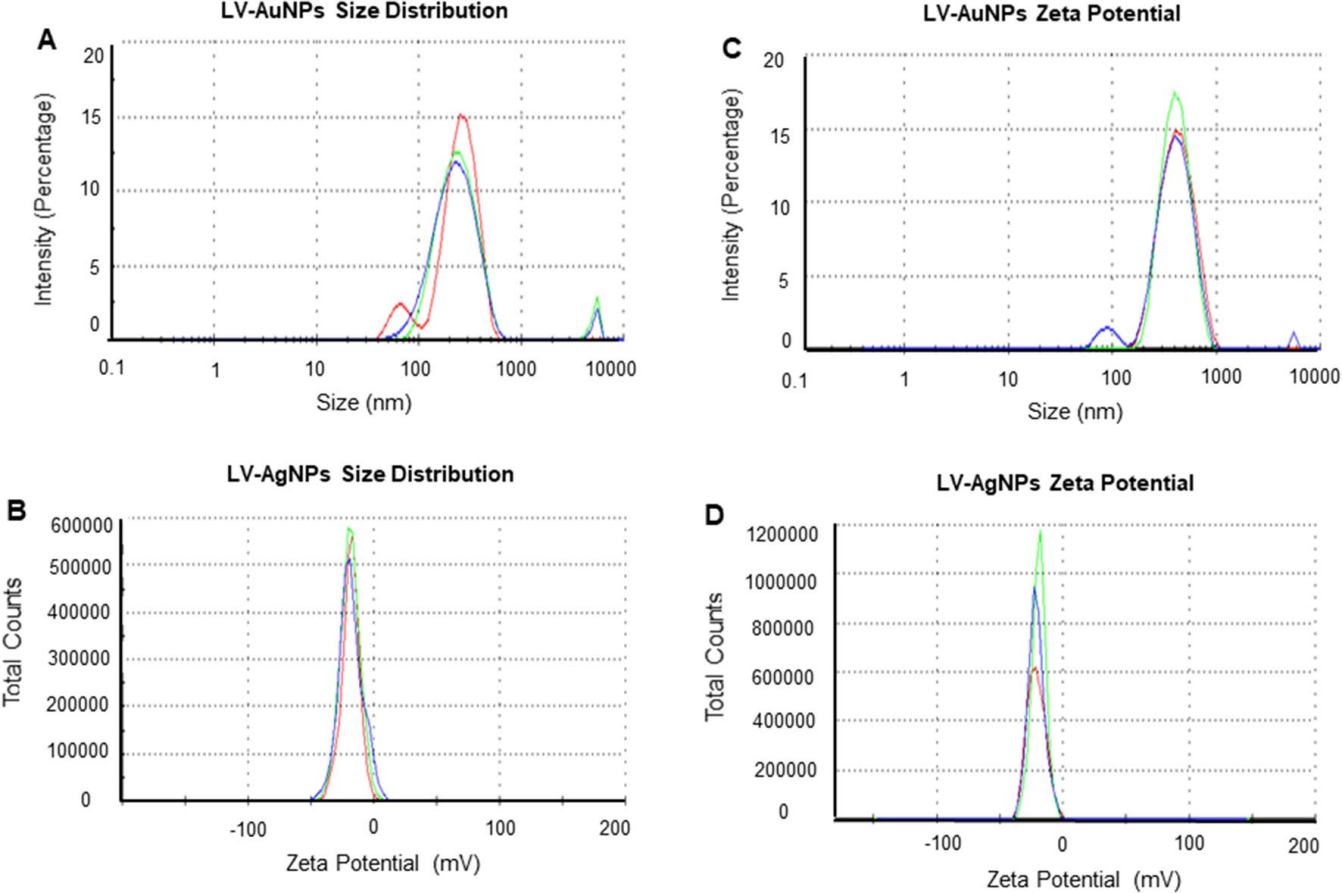
Dynamic light scattering analysis of LV-AuNPs and LV-AgNPs. (A) LV-AuNPs distribution concerning size and intensity (B) LV-AgNPs distribution concerning size and intensity. (C) Zeta potential of LV-AuNPs and (D) LV-AgNPs, representing highly negative surface charge.

The hydrodynamic diameter and zeta potential values of the nanoparticles were then measured using DLS. LV-AuNPs (Fig. 4A) had a size of 292.3 nm and a polydispersity index (PDI) of 0.3, while LV-AgNPs (Fig. 4B) had a size of 542.6 nm and a PDI of 0.479^27^. The zeta potential of as-prepared nanoparticles dispersed in water at ambient temperature showed negative values for LV-AuNPs − 18.3 mV (Fig. 4C) and LV-AgNPs − 20.8 mV (Fig. 4D), indicating negative surface charge^6^.

**Figure 5:**
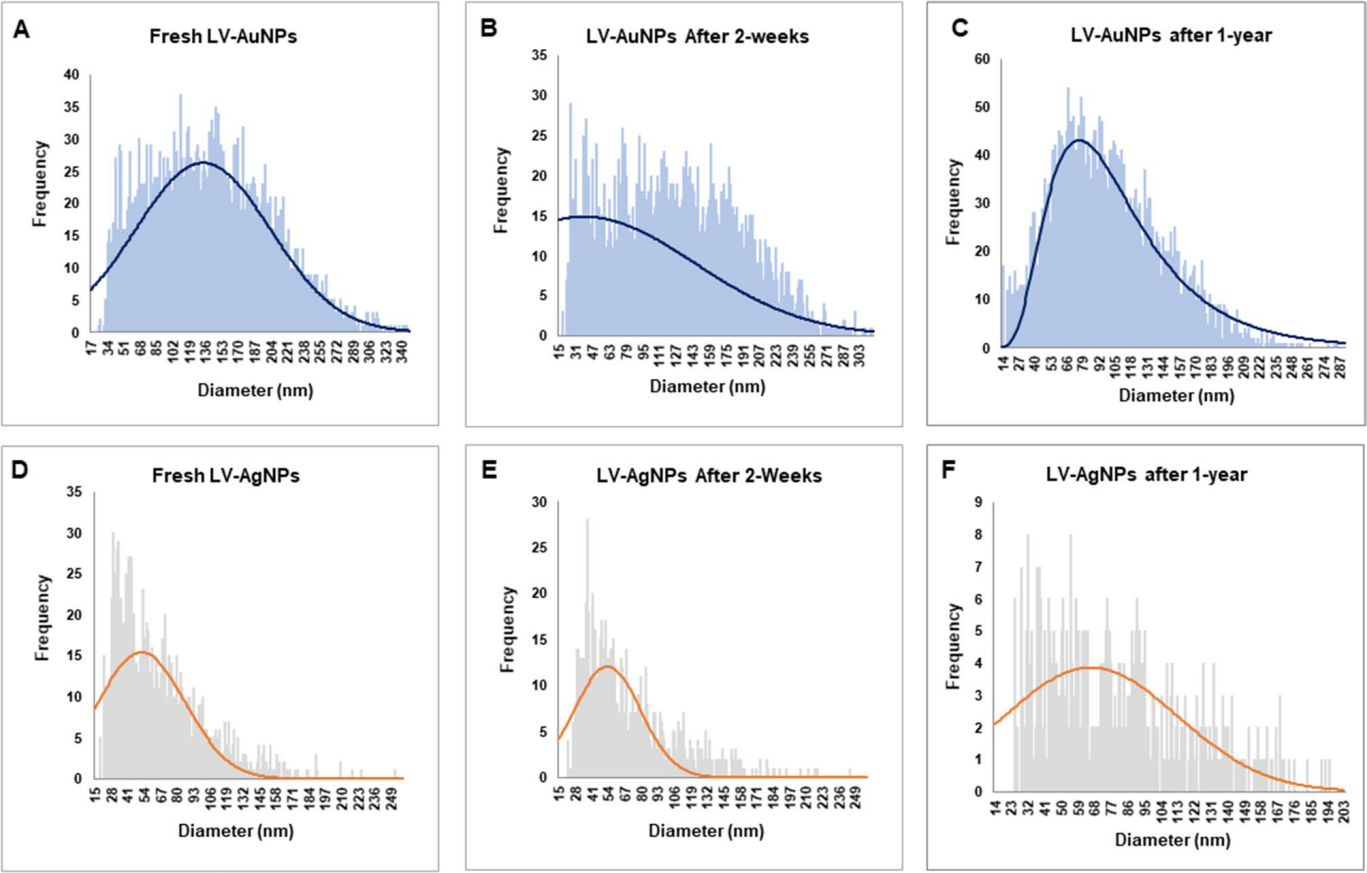
sp-ICPMS and stability analysis of LV-AuNPs and LV-AgNPs. ICPMS histogram of LV-AuNPs at different time intervals (A) fresh LV-AuNPs, (B) after two weeks, (C) after one year at 4 °C. ICPMS histogram of LV-AgNPs at different time intervals (D) fresh LV-AgNPs, (E) after two weeks, (F) after one year at 4 °C.

**Figure 6:**
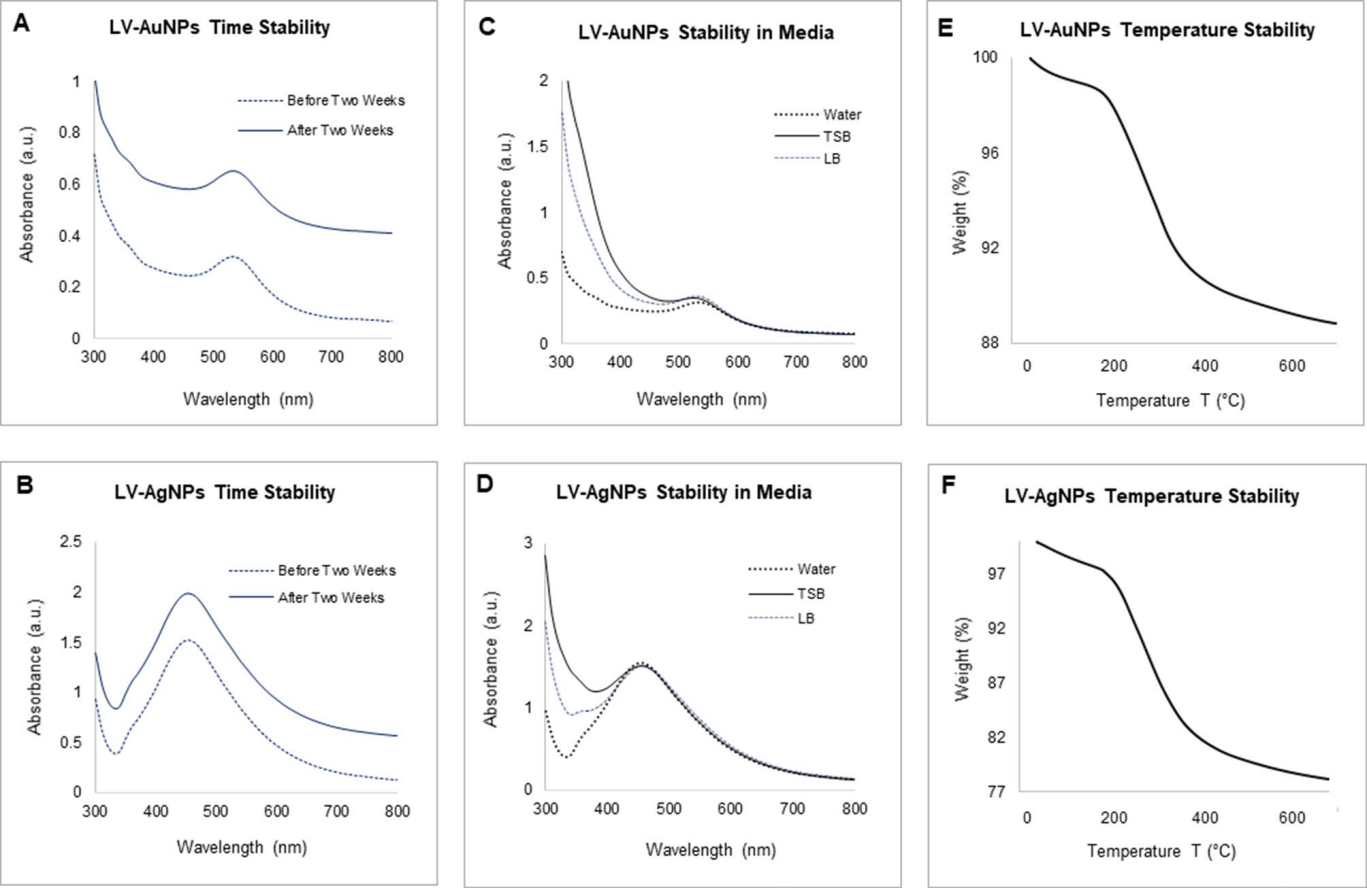
UV-Vis spectrum representing the stability analysis, before and after two weeks of incubation at RT for (A) LV-AuNPs, and (B) LV-AgNPs; in a different medium (C) LV-AuNPs, (D) LV-AgNPs; at the temperature range from 20-700 °C measured by TGA instrument (E) LV-AuNPs, (F) LV-AgNPs.

Next, sp-ICPMS analysis was used to determine the nanoparticle's concentration and stability over time. The concentration of LV-AuNPs was 2.23 g/L (Fig. 5A), while the concentration of LV-AgNPs was 0.615 g/L. (Fig. 5D). After two weeks (Fig. 5B,E) and one year (Fig. 5F), the same samples were tested again (Fig. 5C,F). The collected data showed a consistent histogram with no changes, indicating that the produced nanoparticles remained in the same size and concentration for up to a year, indicating that they were highly stable^20^. UV–Vis analysis was also used to test the stability of nanoparticles at different times and in different mediums. The UV–Vis scan of nanoparticles in a difference of two weeks showed a peak in the same region (Fig. 6A,B). The results of the medium stability test demonstrated that the LV-AuNPs (Fig. 6C) and LV-AgNPs (Fig. 6D) remain stable in all three mediums. Furthermore, TGA was used to conduct the temperature stability, and the results showed complete nanoparticle degradation with increasing temperature (Fig. 6E,F).

**Figure 7:**
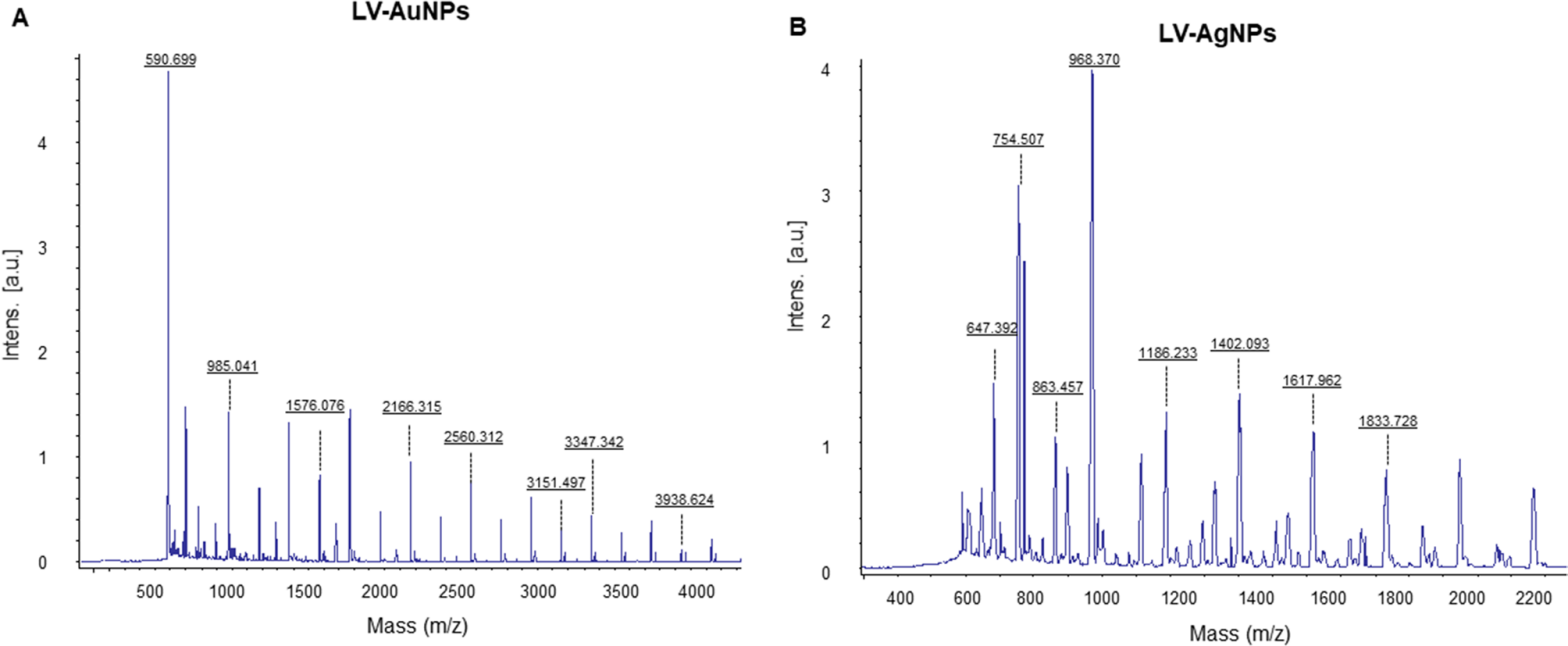
MALDI-TOF analysis of (A) LV-AuNPs and (B) LV-AgNPs demonstrate respective ions.

Furthermore, MALDI-TOF was conducted to examine gold and silver ions on nanoparticle´s surface (Fig. 7A,B). The mass spectra displayed intense single peaks between 590 and 3800 m/z. For LV-AuNPs, several peaks were observed which belong to gold ions, at 590.699, 985.041, 1576.076, 2166.315, 2560.312, 3151.497, 3347.342, and 3938.624 (Fig. 7A). For LV-AgNPs, many peaks were found which could be assigned to silver ions of higher cationic species at 647.392, 754.507, 863.457, 968.370, 1186.233, 1402.093, 1617.962 and1833.728 (Fig. 7B)^5^.

### Antibacterial activity of LV-AgNPs

**Figure 8:**
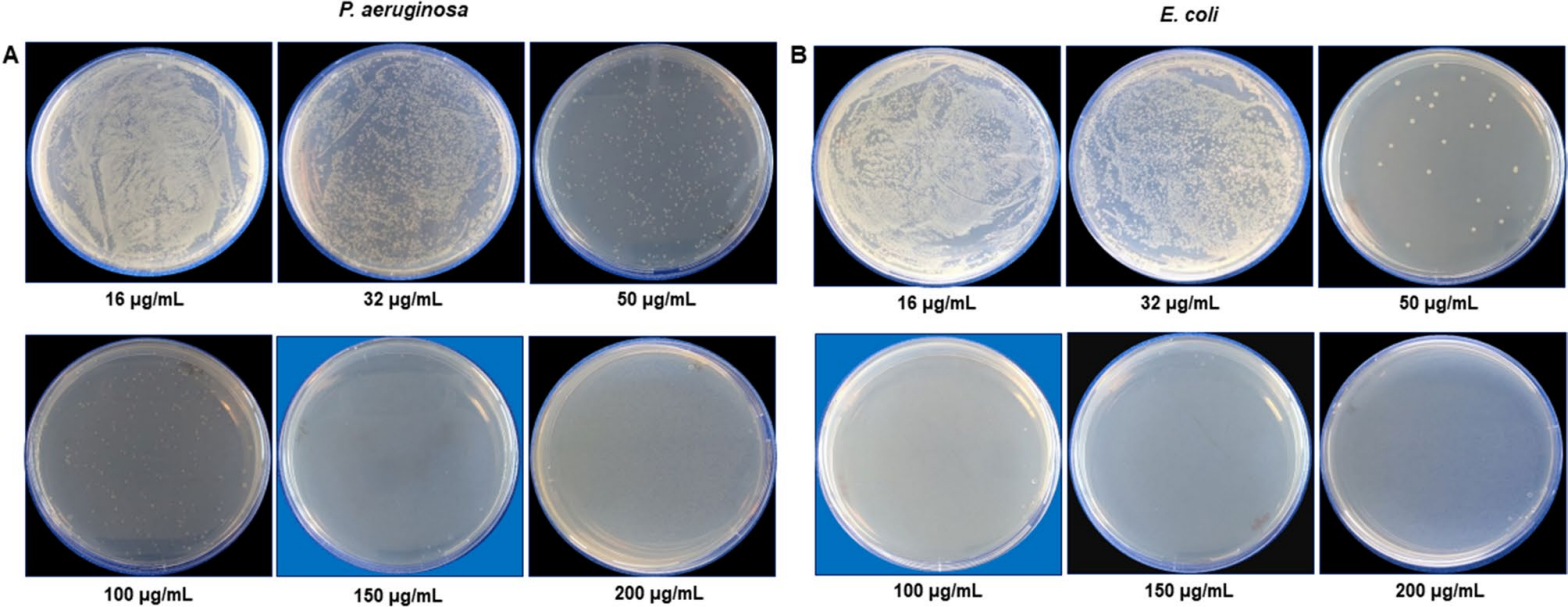
Cell viability test at different concentrations range from 16-200 μg/ml of LV-AgNPs for (A) P. aeruginosa and (B) E. coli. The blue background shows the MBC values of LV-AgNPs against respective pathogens with complete growth inhibition.

**Figure 9:**
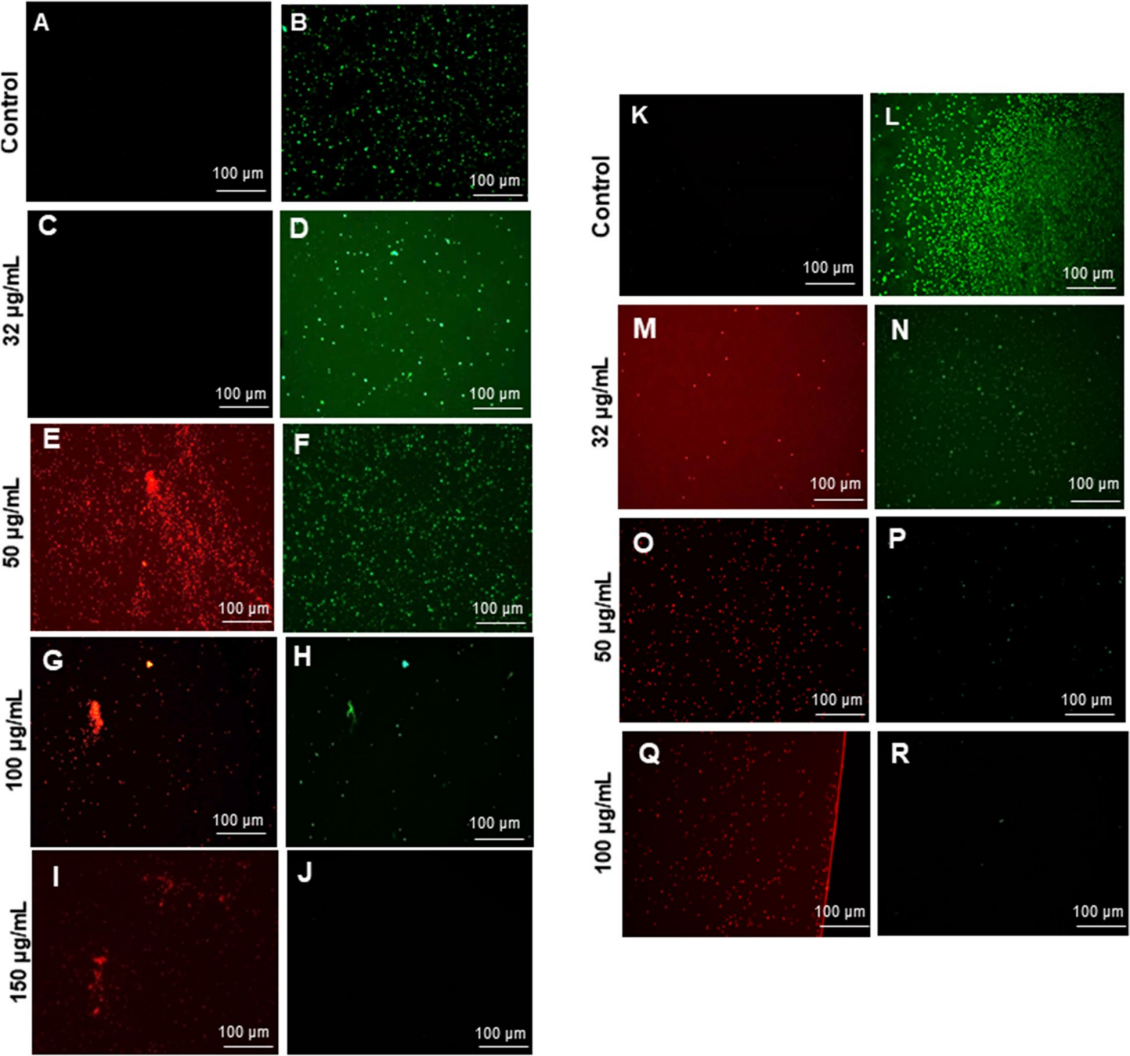
Live and dead staining of (A-J) P. aeruginosa and (K-R) E. coli, after treatment with LV-AgNPs at different concentrations.

LV-AgNPs were explored for the antibacterial activity against *P. aeruginosa* and *E. coli*. LV-AgNPs completely killed the *P. aeruginosa* cells at 150 µg/mL and *E. coli* cells at 100 µg/mL (Fig. 8). The cell viability was further confirmed by using the live and dead staining techniques. *P. aeruginosa* and *E. coli* cells treated with different concentrations of LV-AgNPs were stained with live and dead reagents and observed with a fluorescent microscope (Fig. 9). For *P. aeruginosa* (Fig. 9A–H), many cells were viable at 32–50 µg/mL of LV-AgNPs, but with increasing concentration, the green (live) cells were no longer visible. Concomitantly, the red signal coming from dead cells became increasingly dense, meaning that the LV-AgNPs were toxic to cells and caused the complete death of *P. aeruginosa* cells at 150 µg/mL. Similar results were obtained with *E. coli* cells (Fig. 9I–R), which died completely at 100 µg/mL concentration of LV-AgNPs.

**Figure 10:**
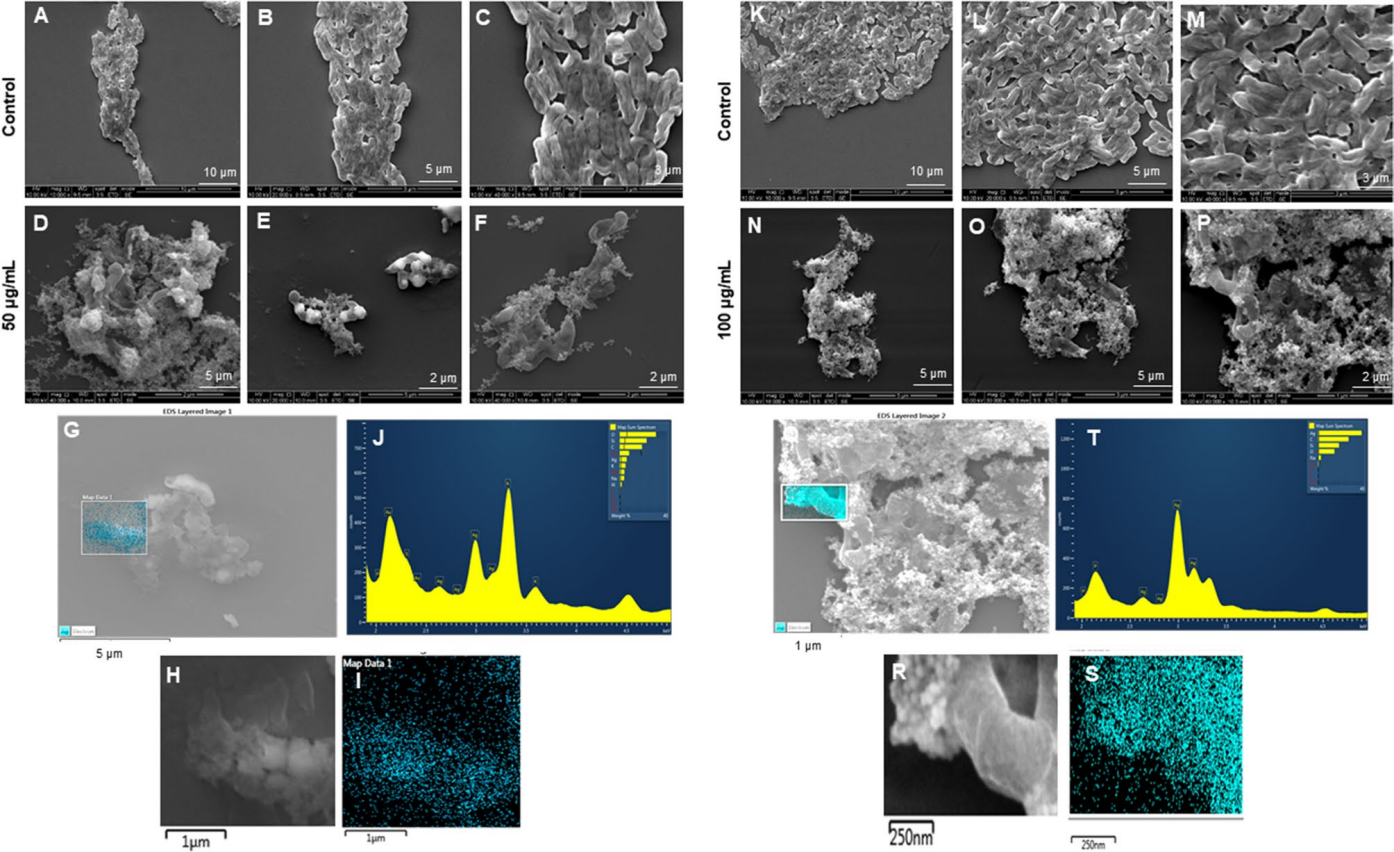
SEM analysis of P. aeruginosa cells after treatment with LV-AgNPs. (A-F) Control cells and LV-AgNPs treated cells with 50 μg/ml at different scales. (G) Scanned image of treated cells (H,I) elemental mapping of the selected area showing silver element in the treated cells, (J) EDX spectrum of the chosen area showing peak for silver element. (K-P) Control cells and LV-AgNPs treated cells with 100 μg/ml at different scales. (Q) Scanned image of treated cells (R,S) elemental mapping of the selected area showing silver element in the treated cells, (T) EDX spectrum of the chosen area showing peak for silver element.

**Figure 11:**
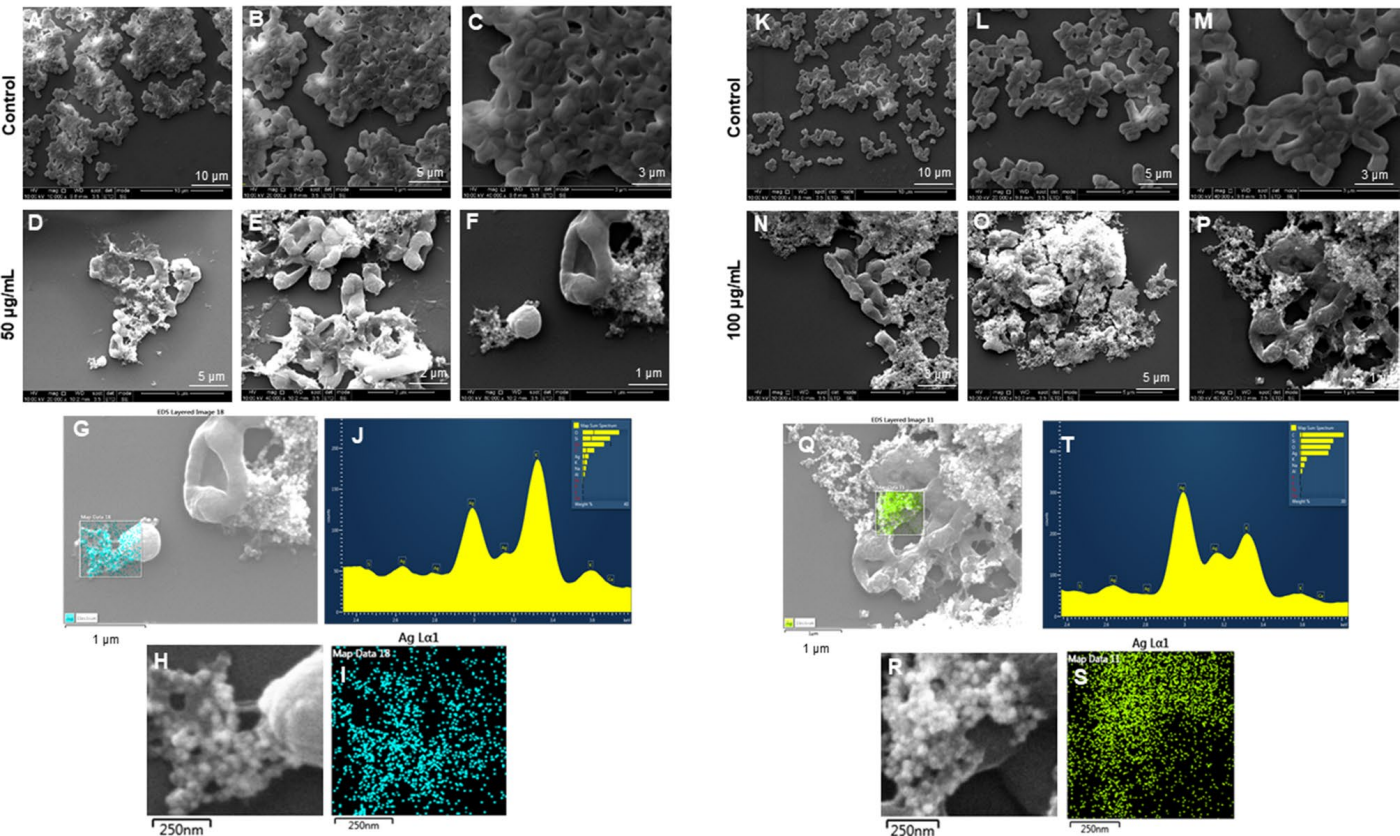
SEM analysis of E. coli cells after treatment with LV-AgNPs. (A-F) Control cells and LV-AgNPs treated cells with 50 μg/ml at different scales. (G) Scanned image of treated cells (H,I) elemental mapping of the selected area showing silver element in the treated cells, (J) EDX spectrum of the chosen area showing peak for silver element. (K-P) Control cells and LV-AgNPs treated cells with 100 μg/ml at different scales. (Q) Scanned image of treated cells (R,S) elemental mapping of the selected area showing silver element in the treated cells, (T) EDX spectrum of the chosen area showing peak for silver element.

SEM was also used to examine the morphology of the dead cells. *P. aeruginosa* cells treated with LV-AgNPs were analyzed by SEM at two concentrations: 50 and 100 g/mL. *P. aeruginosa* cells were totally covered in nanoparticles and had disrupted membrane structures, as seen in Fig. 10D–F. Figure 11N–P shows a similar response at a 100 g/mL concentration of LV-AgNPs. The elemental mapping results also showed that the injured cells were totally covered in LV-AgNPs (Fig. 10G–I,Q–S), confirming that LV-AgNPs were the cause of cell lysis. Furthermore, in injured cells, EDX revealed a clear peak for silver element (Fig. 10J,T), confirming this hypothesis. Figure 11 shows the effects of LV-AgNPs on *E. coli* cells. Pathogenic cells show the open cellular structure with damaged membrane and are covered under the nanoparticles. The EDX and elemental mapping results indicated that the cells were covered predominantly with silver, indicating that the cellular damage occurred due to the silver ions released from LV-AgNPs Fig. 11G–I,Q–S.

## Discussion

Our method uses the aqueous extract of *L. vulgare* berries to reduce gold and silver salt into nanoparticles. For both LV-AuNPs and LV-AgNPs, the dark purple and deep brown color generated in the SM is a clear sign of the nanoparticle's appearance. The nanoparticles were purified and displayed again overlapping absorbance with higher intensity in the UV–Vis spectrum. Optimization studies for LV-AuNPs, demonstrated that the diluted berries extract (1:20 dilution) offers the best conditions for LV-AuNPs production. With such high dilutions, it is possible to produce many gold nanoparticles using very little biological resources. Additionally, the process did not require any additional reducing or stabilizing agents; the aqueous extract was enough to do the job. The gold salt optimization study revealed an optimum peak at 2 mM concentration. Any further increase in salt level causes peak shifting and broadening, which means that the nanoparticles lost their structures by precipitating. The optimum reduction time absorbed was 5 min for the time performance case. Keeping the SM under the same conditions caused nanoparticles to settle, as clearly visible in Fig. 2C. Until five minutes, nanoparticles solution appeared to be homogenous, and peaks also showed a fine and narrow appearance. For the LV-AgNPs optimization study, 1:10 (100 µL) of extract diluted with water displayed optimum production, which is advantageous because synthesis used very few amounts of resources. The temperature study showed 90 ℃ is the optimum temperature with the highest peak in UV–Vis absorbance and homogeneity in solution Fig. 2E. 5 mM of silver salt came out as the optimum salt concentration for completely reducing silver salt to nanoparticles within 15 min. All the optimization study UV–Vis peaks align with the visible picture of SMs shown below each graph.

The structural morphology of nanoparticles showed a big difference between the TEM and DLS size analysis, especially for LV-AgNPs. It is well known that the size difference between the two methods is due to the fact that TEM shows core nanoparticles size and DLS shows hydrodynamic diameter. However, despite the two values being overall correlated, the big difference most likely reflects a large amount of biological components around the surface of the nanoparticles, which form the corona layer. For any nanoparticles applications, stability is the utmost requirement ^28^. The corona layer helped our nanoparticles to maintain their structure and stability for more than one year in different media and solutions, as shown in the ICPMS study (Fig. 5) and stability analysis study (Fig. 6). ICPMS pattern of LV-AuNPs displays a similar distribution of nanoparticles between 17 and 250 nm in a difference of two weeks and after one year (Fig. 5A–C). LV-AgNPs also exhibited a similar behavior by showing the maximum frequency of nanoparticles in size range 15–100 nm for fresh samples and keeping the nanoparticles solution for two weeks and one year (Fig. 5D–F). UV–Vis spectrum-based analysis of two weeks incubation and incubation in different media also revealed overlapping patter in peaks, which means nanoparticles were not affected by changing media solutions or time. This high level of stability was achieved due to the surrounding corona layer that forms during synthesis without adding any additional stabilizer and remains with nanoparticles for a prolonged period and at ambient conditions^29^. In addition, the negative zeta potential value also showed that the nanoparticles are stabilized by electrostatic repulsion and steric hindrance of organic moieties present in the *L. vulgare* berries extract. The TGA graph shows that the nanoparticles are degradable at higher temperatures despite the corona layer. From 200 to 400 ℃, the corona layers start depleting slowly and further increase in temperature more than 400 ℃ causes complete nanoparticles destruction.

Any bioactive property exhibited by nanoparticles depends on their physical properties, such as shape and size, which are largely determined by the conditions of synthesis^30^. LV-AgNPs were explored for antibacterial study against *P. aeruginosa* and *E. coli.* Results showed an MBC value of 150 µg/mL against *P. aeruginosa* and 100 µg/mL against *E. coli*, higher than the values reported in previous studies where similar systems were explored. For instance, Patra et al. showed the MBC value of silver nanoparticles produced using corn leaf waste of *Zea mays*, as 100 µg/mL against *E. coli*^31^, similar to our case. Loo et al. showed the MBC value of silver nanoparticles from pu-erh tea leaves extract against *E. coli* as 7.8 µg/mL^32^. Khorrami et al. demonstrated the walnut husk mediated silver nanoparticles MBC value against *P. aeruginosa* and *E. coli* as 20 and 5 µg/mL^33^. We concluded that the weak antimicrobial activity (high MBC values) compared to the examples mentioned above could be due to the think corona layer, which provides less toxicity butlong-term stability. The extreme thickness of corona could be judged by the size difference between TEM (20–70 nm) and DLS (542.6 nm) for LV-AgNPs. The membrane is also visible in the TEM image of LV-AgNPs (Fig. 3S,T). The live and dead staining experiment also followed the similar pattern of plate assay results and showed maximum green cells (live) at 32 µg/mL and 50 µg/mL in *P. aeruginosa;* however, the green intensity reduces and red increases at 100 and 150 µg/mL, which means more cells are dead than alive. For *E. coli,* at 50 and 100 µg/mL, most cells appeared to be dead due to LV-AgNPs effects.

The SEM morphological analysis of treated cells displayed the complete open structure of individual cells with the nanoparticles surrounding them (Fig. 10D–F,N–P). The image demonstrates that cells died due to the lysis and membrane damage caused by LV-AgNPs action. The small size of LV-AgNPs could also help the nanoparticles internalize and damage the other cellular structures, such as DNA, proteins, ribosomes, etc.^34,35^. The other possibility of cell death is the generation of reactive oxygen species (ROS) once the nanoparticles are internalized, a well-known mechanism for the silver nanoparticles' antimicrobial effects^30,36^. Elemental mapping scanning of the nanoparticles surrounded cells also showed a clear and sharp peak for silver elements in the EDX spectrum and distribution (blue) in mapping analysis (Fig. 10G–I,Q–S). Comparatively, the control cells remain intact without any damage. Figure 11D–F,N–P displayed the *E. coli* cells damaged and formed hollow structures, which resemble complete lysis due to the LV-AgNPs action at two concentrations 50 and 100 µg/mL. The porins present in the membrane of Gram-negative bacteria are large enough to allow the passive diffusion of small nanoparticles. This represents another possibility to allow nanoparticles uptake by the cells, leading to internal damage in addition to membrane leakage. The destruction caused by silver ions released from nanoparticles was further confirmed by the elemental mapping and EDX analysis, which resemble silver element distribution and sharp peak at 3 keV (Fig. 11G–I,Q–S). Our reports are in alignment with previously published results.

The important and noticeable feature in the antimicrobial study of nanoparticles is that the LV-AgNPs remain intact in their appearance after killing the pathogenic cells. Figure 10F,O,P for *P. aeruginosa* cells and Fig. 11D,F,O,P for *E. coli* cells shows the dead cells with open structures with spherical silver nanoparticles surrounding them. This shows that nanoparticles remain stable even after delivering their action either intracellularly or extracellularly and manage to come out of cells after complete lysis. Again, the thick corona layer helps them maintain their identity without losing any stability. The question is would it be possible to retrieve these nanoparticles and apply them again for a similar purpose. Since the nanoparticle came in contact with a living organism that changed the corona composition, would the LV-AgNPs with the new corona layer have the same effect? These questions are remained to answer in future research and probably solve the doubts about the reusability of nanoparticles and their environmental impact.

## Conclusions

We used *L. vulgare* berries for the efficient production of gold and silver nanoparticles in a rapid, efficient, and economical method. *L. vulgare* berries are mostly not used for any purpose and hence considered a waste. This study demonstrates the use of a waste resource to produce a value-added product. *L. vulgare* berries to manufacture commercial nanoparticles will assist in making the process more sustainable by lowering costs and repurposing waste. The results showed that the biological components from *L. vulgare* berries extract played a critical role in completely reducing and stabilizing nanoparticles by forming a transparent layer around them. LV-AgNPs showed antimicrobial potential with an MBC value of 150 µg/mL against *P. aeruginosa* and 100 µg/mL against *E. coli.*
